# The usage of drainage after primary total hip or knee arthroplasty: best evidence selection and risk of bias considerations

**DOI:** 10.1186/s12891-021-04897-z

**Published:** 2021-12-08

**Authors:** Huibin Long, Zhichang Li, Dan Xing, Yan Ke, Jianhao Lin

**Affiliations:** 1 Department of Orthopedics, Beijing Friendship Hospital, Capital Medical University, Beijing, China; 2 Arthritis Clinic & Research Center, Peking University People’s Hospital, No. 11 South Street of Xizhimen, Xicheng District, Beijing, 100044 China; 3 Arthritis Institute, Peking University, Beijing, China

**Keywords:** Arthroplasty, Overview, Drainage, Evidence, Risk-of bias

## Abstract

**Background:**

Numerous systematic reviews investigating the benefit of the usage of drainage after primary total hip or knee arthroplasty have been published with divergent conclusions. We aim to determine the best available evidence and consider risk of bias of these articles and to provide recommendations.

**Methods:**

A systematic search of systematic reviews published through to May 2020 was performed in MEDLINE, EMBASE and Cochrane library. Methodological quality, risk of bias and best evidence choice of included articles were evaluated by AMSTAR instrument, ROBIS tool and Jadad decision algorithm, respectively. We selected systematic reviews with high methodological quality and low risk of bias ultimately as best evidence.

**Results:**

Twelve meta-analyses were included lastly. According to the ROBIS tool, seven of the included systematic reviews were with low risk of bias and five with high risk of bias. The Jadad decision algorithm suggested that two reviews conducted by Zan et al. for hip and Si et al. et al. for knee were selected as the best evidence, with highest AMSTAR score and low risk of bias.

**Conclusions:**

Ten systematic reviews were included as low-quality with only two high-quality studies. Based on the current available evidence, we have insufficient confidence to draw conclusion that whether to use closed suction drainage for both total knee and hip arthroplasty. To verify the necessity and benefit of using closed suction drainage after primary total knee and hip arthroplasty, and develop exact recommendations, further studies are still required.

**Supplementary Information:**

The online version contains supplementary material available at 10.1186/s12891-021-04897-z.

## Background

In order to prevent the formation of hematoma and therefore reduce the incidence of related complications including infection, a closed suction drainage is routinely installed at the end of orthopaedic surgeries since at least 60 years ago [1]. But the necessity and benefit of its usage were firstly doubted by Reilly 25 years later [2]. With the popularity and recognition of Fast Track and Enhanced Recovery After Surgery (ERAS), more and more surgeons have tried to abandon this ‘routine’ procedure [3], especially after the American Academy of Orthopaedic Surgeons guideline on surgical management of knee osteoarthritis was released, which recommended not to use drainage after total knee arthroplasty (TKA) with “Strong Evidence” [4].

Evidence-based medicine has obtained recognition and popularity with the purpose to provide best selection in clinical practice since last decade [5]. Although numerous meta-analyses or systematic reviews have been published to evaluate the necessity and benefit of the usage of drainage after primary total hip or knee arthroplasty [6–17], the pooled conclusions were still discordant and could not provide more potent evidence. Thus, it is difficult for clinical professionals to determine whether to use drainage after total hip or knee arthroplasty based on the conflicting conclusions of these systematic reviews.

We therefore put forward three objectives of the present study: (1) to conduct a summary of systematic reviews investigating the necessity of the usage of drainage after total hip or knee arthroplasty; (2) to assess the quality of methodology and risk of bias of included systematic reviews and (3) to determine which systematic review provide evidence qualitatively and recommendations for the usage of drainage.

## Materials and methods

### Search strategy

All meta-analyses or systematic reviews published through to May 2020 that fulfilled the following inclusive criteria were searched in databases including MEDLINE, EMBASE and Cochrane library. The literature procedure was performed using the guideline of Preferred Reporting Items for Systematic Reviews and Meta-analysis (PRISMA) statement [18], which was considered to meet high-quality reporting demand of meta-analyses or systematic reviews [19]. The MeSH words and free items used to assess the exactness of search strategy included: “drain”, “drainage”, “arthroplasty”, “replacement”, “hip”, “knee”, “systematic review” and “meta-analysis”. The citations of potentially included articles were also screened to ensure no relevant articles were missed. Two authors did this independently.

### Inclusive and exclusive criteria

Primary studies were considered eligible for inclusion if they met the following criteria: Meta-analyses or systematic reviews evaluating the outcomes of total knee or hip arthroplasty with closed suction drainage, comparing with the outcomes without closed suction drainage.

Exclusion criteria included: Papers of abstract, commentary, methodological study, narrative review, overview, not written in English.

### Study selection

Firstly, two trained reviewers independently screened the titles and abstracts of potential articles following the inclusive criteria. Both reviewers were blinded to the names of researchers, institutions and journals of potential included studies. To take the final inclusion decision, the full text of the primary articles that potentially met the inclusive criteria was assessed. Any disagreement was settled after discussion to reach a consensus or a third reviewer was involved.

### Data extraction

Data from the included studies were extracted by two trained reviewers independently under the application of a standard data extraction form. Items including title, authors, original study design, searched database, total number of included studies, level of evidence, the pooled results and methodological variables were extracted.

### Methodological quality appraisal

Two reviewers independently performed the methodological quality assessment, and any controversial conclusions were settled by discussion or consulting a third reviewer. The Assessment of Multiple Systematic Reviews (AMSTAR) method [20] was used to evaluate the methodological quality of included meta-analyses or systematic reviews. AMSTAR was a methodological measurement tool demonstrated to have perfect validity, reliability and responsibility [21], and containing 11 items for appraisal of methodological quality of published meta-analyses and systematic reviews [22].

### Heterogeneity assessment

Heterogeneity results of each outcome were extracted from the included systematic reviews when with pooling results. We also evaluated that whether the possible sources of heterogeneity within primary original studies were considered and whether the authors performed sensitivity analysis. As stated in the Cochrane Handbook, heterogeneity between 0 and 40% is considered as not important; between 30 and 60% as moderate; between 50 and 90% as substantial, and between 75 and 100% as considerable. Ultimately, *I*^2^ value was applied to determine the degree of heterogeneity quantitatively and *I*^2^ less than 60% was accepted in the present study.

### Best evidence choice

The procedure of best evidence choice was performed according to the Jadad decision algorithm [23], which was aimed to help to select decisive articles. Sources of inconsistency among meta-analyses included: clinical question, inclusion and exclusion criteria, data extraction, quality assessment, data pooling, and statistical analysis. The methodological instrument determines the above sources of discordances [23]. Two trained authors applied the algorithm instrument independently. We came to conformity in the present study as to which of the included studies can provide the best available evidence.

### Risk of bias assessment

With the help of ROBIS tool [24], the risk of bias assessment for included systematic reviews was performed. Disagreements were resolved by discussion or involving a third reviewer. Under the guidance of the ROBIS tool, we evaluated the risk of bias by assessing four domains: study inclusive criteria, recognition and selection of studies, data collection and study assessment, and synthesis and findings. The above four domains covered the main processes of review.

Information that adopted to sustain the judgments, signaling questions, and judgment of concern about risk of bias was assessed for each domain. The answers for the signaling questions included: ‘Yes’, ‘Probably Yes’, ‘No’, ‘Probably No’ and ‘No Information’. Answer only with ‘Yes’ reveals low concerns. Thus, ‘Low’, ‘High’, or ‘Unclear’ was concluded for risk of bias of each domain. While all signaling questions for the domain were ‘Yes’ or ‘Probably Yes’, the domain was classified as low level of concern. Once any signaling questions were reported as ‘No’ or ‘Probably No’, concern about risk of bias was raised [24].

## Results

### Literature search

**Fig. 1:**
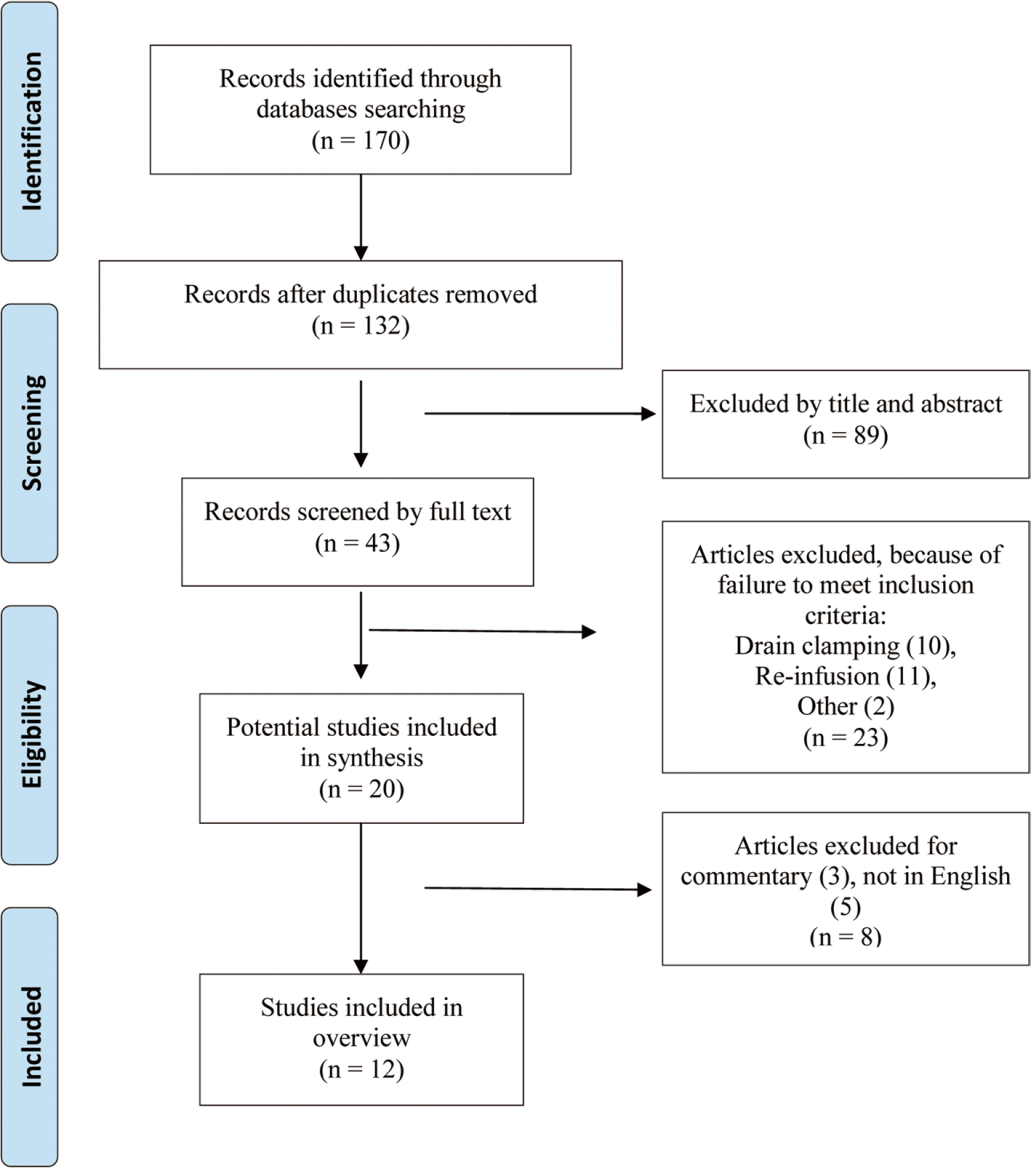
Flowchart of the study selection process

After duplicates were removed following the search strategy, a total of 132 titles and abstracts were preliminarily identified, of which 12 of the issued systematic reviews [6–17] met the inclusive criteria ultimately (Fig. 1). Table 1 showed the characteristics of included studies. The number of primary original studies varied from 3 in the study published in 2015 [13] to 20 that published in 2013 [10] (Supplementary Table 1). All included systematic reviews conducted qualitatively data synthesis. Four reviews included only hip surgery and five only included knee. Two Cochrane reviews [6, 8] included all the orthopedic surgeries and one systematic review [7] included both hip and knee surgery. We extracted data of hip and knee arthroplasty separately from these three reviews.

**Table 1:** General Description of the Characteristics of included Systematic Reviews

Authors	Journal	Date of Last literature search	Date of Publication	No. of included studies	No. of gray literature
Parker et al. 2001	Cochrane Database of Systematic reviews	May-01	Oct-01	2/12/21	0
Parker et al. 2004	J Bone Joint Surg Am	Mar-03	Jun-04	18	0
Parker et al. 2007	Cochrane Database of Systematic reviews	Mar-06	Jul-07	2/19/36	0
Zhang et al. 2011	J Arthroplasty	May-10	Dec-11	15	0
Zhou et al. 2013	Int Orthop	Dec-12	Aug-13	2/20	0
Chen et al. 2014	Eur J Orthop Surg Traumatol	May-13	Aug-13	16	0
Kelly et al. 2014	Surg Technol Int	NM	Mar-14	16	0
Li et al. 2015	J Orthop Surg Res	May-14	Jan-15	3	0
Quinn et al. 2015	Int Orthop	Nov-12	Jul-14	6	0
Si et al. 2016	BMC Musculoskelet Disord	Feb-16	Apr-16	12	0
Zan et al. 2016	Int J Clin Exp Med	Jul-15	Feb-16	12	0
Zhang et al. 2018	Medicine	Jun-17	Jun-18	19	0

### Search methodology

Details of the search strategy which was applied by included systematic reviews were showed in Table 2. Medline, Embase and Cochrane Library are the most frequency source of the included meta-analyses.

**Table 2:** Databases Mentioned by Included Systematic Reviews during Literature Searches

Authors	Search Database								
	Medline	Embase	Cochrane	BIOSIS	EBSCO	Google	CINAHL	CENTRAL	Others
Parker et al. 2001	**+**	**–**	**+**	**–**	**–**	**–**	**–**	**–**	**–**
Parker et al. 2004	**+**	**+**	**+**	**–**	**–**	**–**	**+**	**+**	**+**
Parker et al. 2007	**+**	**+**	**+**	**–**	**–**	**–**	**+**	**+**	**+**
Zhang et al. 2011	**+**	**+**	**+**	**–**	**–**	**–**	**–**	**–**	**+**
Zhou et al. 2013	**+**	**+**	**+**	**–**	**–**	**–**	**–**	**–**	**–**
Chen et al. 2014	**+**	**+**	**+**	**–**	**–**	**–**	**–**	**–**	**+**
Kelly et al. 2014	**+**	**–**	**–**	**–**	**–**	**–**	**–**	**+**	**+**
Li et al. 2015	**+**	**+**	**+**	**–**	**–**	**+**	**–**	**–**	**+**
Quinn et al. 2015	**+**	**+**	**+**	**–**	**–**	**–**	**+**	**–**	**–**
Si et al. 2016	**+**	**+**	**–**	**–**	**–**	**–**	**–**	**+**	**+**
Zan et al. 2016	**+**	**+**	**+**	**–**	**–**	**+**	**–**	**–**	**+**
Zhang et al. 2018	**+**	**+**	**+**	**–**	**–**	**–**	**–**	**–**	**–**

### Methodological quality

Methodological characteristics of included studies are presented in Table 3. All studies included prospective randomized trials (RCTs) or quasi-randomized trials (qRCTs) and were Level II of evidence. Only the two Cochrane reviews included prospective trials in which the treatment allocation was inadequately concealed and were Level III of evidence. We used REVMAN and STATA software in meta-analyses with pooling data. Subgroup analyses (between the drainage and non-drainage groups) were performed in six of the included meta-analyses [6–8, 15–17]. One systematic review [11] used GRADE in their study. Four studies [9–11, 16] performed sensitivity analysis. The AMSTAR instrument with all question items for each systematic review are shown in Table 4. AMSTAR scores ranged from 6 to 10 with an average score of 7.8. The systematic review conducted by Li et al. [13] was of the highest quality.

**Table 3:** Methodological Characteristics of Included Systematic Reviews

Authors	Primary Study design	Level of evidence	Software	Sensitivity analysis	Subgroup analysis	GRADE evidence profiles
Parker et al. 2001	Prospective trials	**II/III**	**NM**	**No**	**Yes**	**No**
Parker et al. 2004	RCT and qRCT	**II**	**NM**	**No**	**Yes**	**No**
Parker et al. 2007	Prospective trials	**II/III**	**NM**	**No**	**Yes**	**No**
Zhang et al. 2011	RCT and qRCT	**II**	**Revman**	**Yes**	**No**	**No**
Zhou et al. 2013	RCT	**II**	**Revman and Stata**	**Yes**	**No**	**No**
Chen et al. 2014	RCT and qRCT	**II**	**Revman**	**Yes**	**No**	**Yes**
Kelly et al. 2014	RCT and qRCT	**II**	**Revman**	**No**	**No**	**No**
Li et al. 2015	RCT	**II**	**Revman**	**No**	**No**	**No**
Quinn et al. 2015	RCT	**II**	**Revman**	**No**	**No**	**No**
Si et al. 2016	RCT	**II**	**Revman**	**No**	**Yes**	**No**
Zan et al. 2016	RCT	**II**	**Revman**	**Yes**	**Yes**	**No**
Zhang et al. 2018	RCT	**II**	**Stata**	**No**	**Yes**	**No**

*NM* not mentioned, *RCT* randomized controlled trials, *qRCT* quasi-randomized controlled trials

**Table 4:** AMSTAR Criteria for Included Systematic Reviews

Items	Parker et al. 2001	Parker et al. 2004	Parker et al. 2007	Zhang et al. 2011	Zhou et al. 2013	Chen et al. 2014	Kelly et al. 2014	Li et al. 2015	Quinn et al. 2015	Si et al. 2016	Zan et al. 2016	Zhang et al. 2018
Was a prior design provided?	1	0	1	0	0	0	0	0	0	0	0	0
Was there duplicate selection and data extraction?	1	1	1	1	1	1	1	1	1	1	1	1
Was a comprehensive literature search preformed?	1	1	1	1	1	1	0	1	1	1	1	1
Was the status of publication used as an inclusion criterion?	1	1	1	1	1	1	1	1	1	1	1	1
Was a list of included/excluded studies provided?	1	1	1	0	0	0	0	1	1	0	0	0
Were the profiles of the included studies provided?	1	1	1	1	1	1	0	1	1	1	1	1
Was the methodological quality of the included studies evaluated and documented?	1	1	1	1	1	1	1	1	1	1	1	1
Was the scientific quality of the included studies used appropriately in formulating conclusions?	1	1	1	1	1	1	1	1	1	1	1	1
Were the methods used to combine the findings of studies appropriate?	0	0	0	0	0	0	0	1	0	0	0	0
Was the publication bias evaluated?	0	0	0	0	0	1	1	1	0	0	1	0
Were the conflicts of interest stated?	1	1	1	1	1	1	1	1	1	1	1	1
Total score	9	8	9	7	7	8	6	10	8	7	8	7

### Heterogeneity assessment

Heterogeneity results of each outcome with pooled quantitatively in the included systematic reviews have been listed in Supplementary Table 2. *I*^2^ statistic value was assessed as a method to showed the study heterogeneity among studies.

### Jadad decision algorithm

**Fig. 2:**
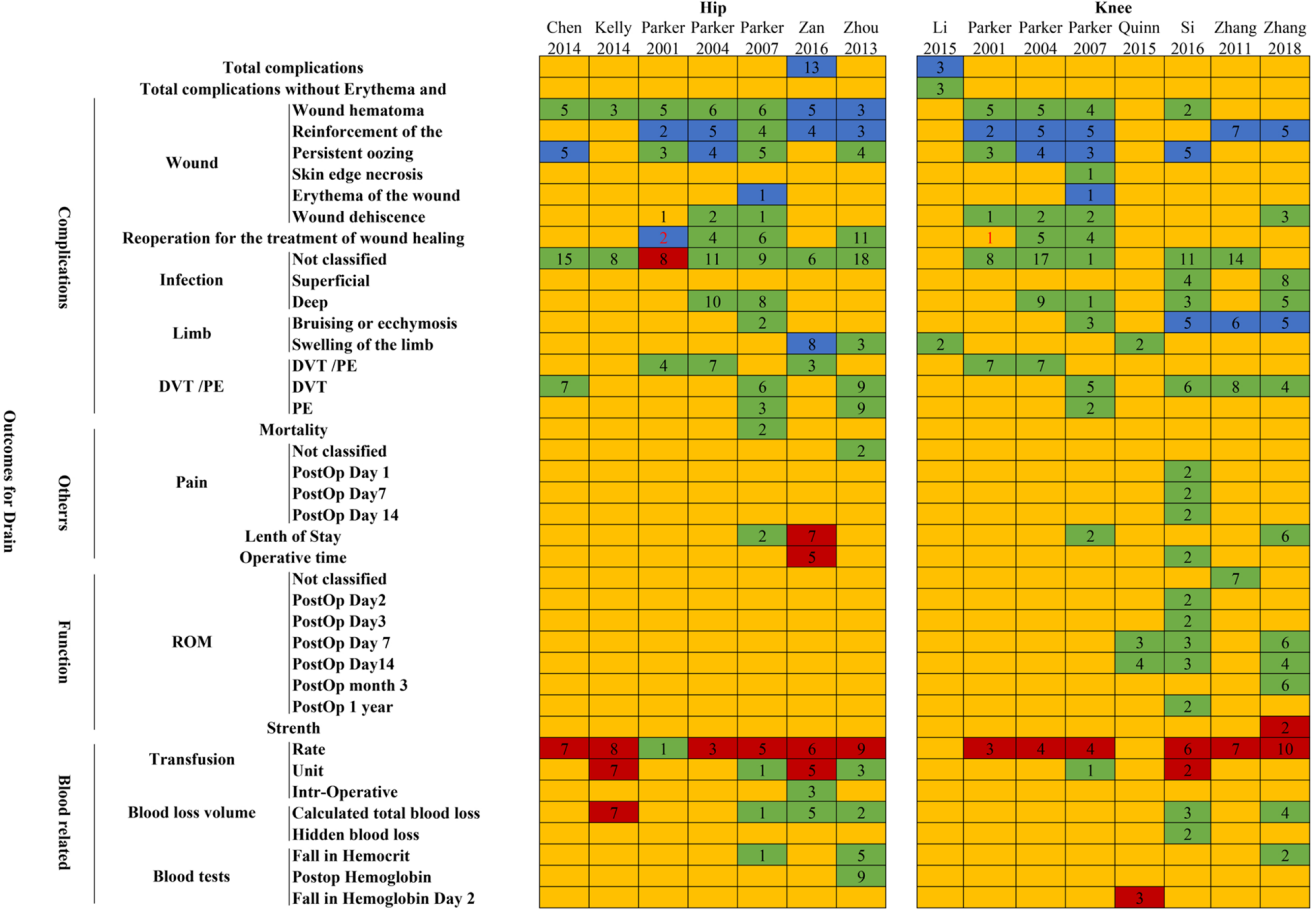
Results of each included systematic review. Red means favoring No-Drainage; green means no difference; yellow means not reporting; and blue means favoring Drainage. Arabic numerals mean the number of included randomized clinical trials

**Fig. 3:**
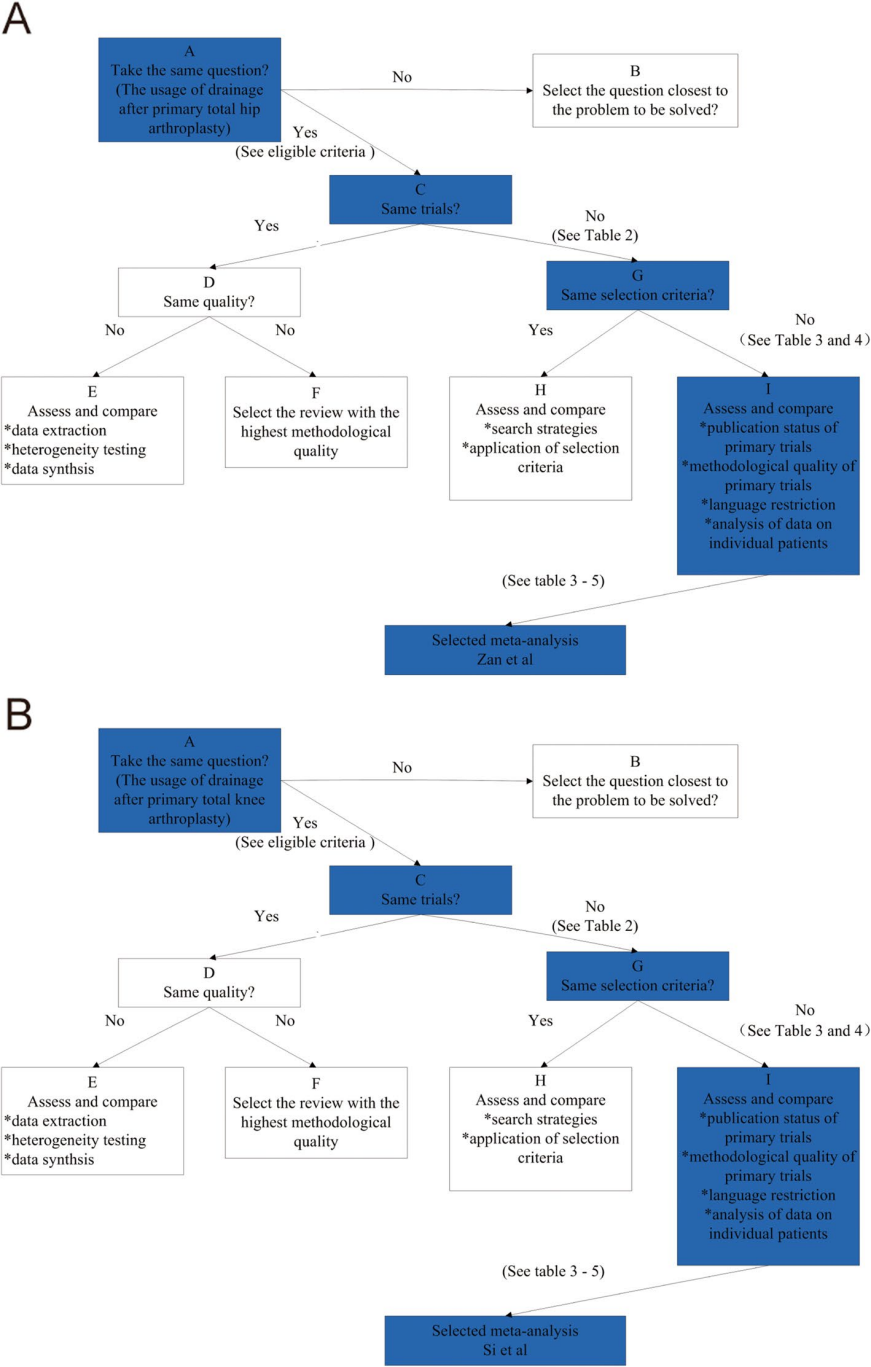
Flow diagram of Jadad decision algorithm

To determine which of the included articles offered the best evidence to the usage of drainage after primary total hip or knee arthroplasty, the Jadad decision algorithm was performed. All variables reported in the included articles were presented in Fig. 2. According to the procedure of Jadad decision algorithm (the same clinical question addressed by systematic reviews, not include all the duplicate primary trials, not have similar inclusive criteria), the craved systematic reviews can be selected based on the methodological quality and publication stature (Fig. 3). As most systematic reviews evaluated hip or knee arthroplasty only, we did the Jadad decision algorithm separately for hip and knee arthroplasty. Two systematic reviews with highest quality were selected ultimately, Zan et al. [16] for hip and Si et al. [15] for knee.

### Risk of bias

**Fig. 4:**
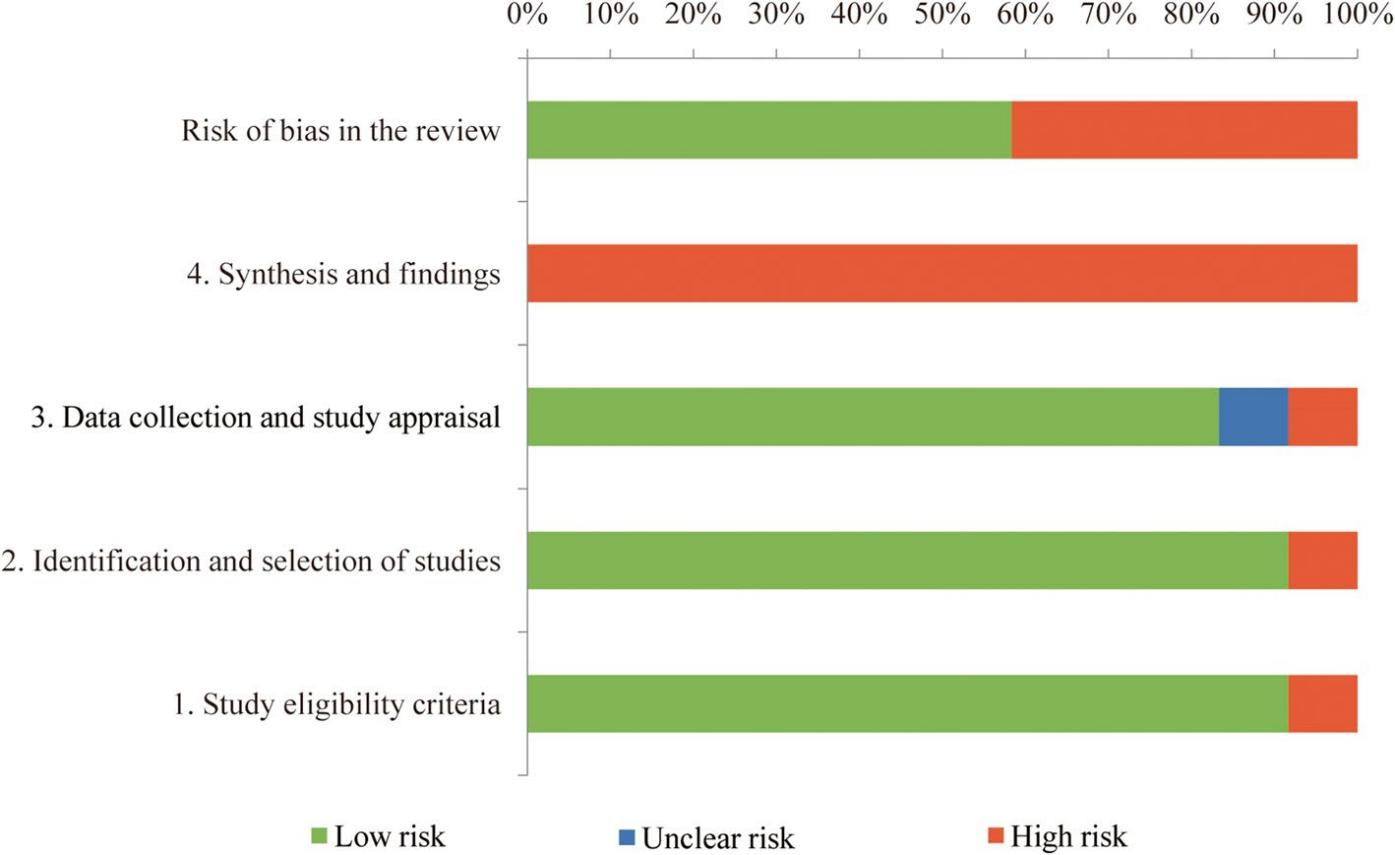
Risk of bias of the included systematic reviews with ROBIS tool

Table 5 showed the results at risk of bias of eligible studies evaluated by ROBIS. We also included the appraisal results in phase 2 at each item of ROBIS. The 3rd phase indicated conclusions at risk of bias on the systematic reviews. Seven studies [9–11, 13, 15–17] were at low risk of bias, while the other five at high risk of bias [6–8, 12, 14]. Figure 4 showed the judgements regarding each item of ROBIS as percentages through all the eligible studies. To provide best evidence, we selected two systematic reviews [15, 16] with higher methodological quality and lower risk of bias based on the AMSTAR instrument and ROBIS tool.

**Table 5:**
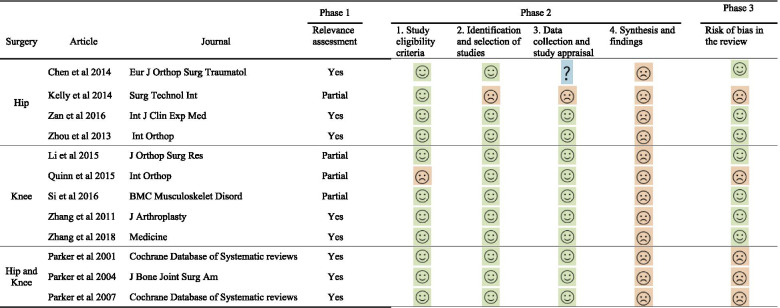
Risk of bias assessment of systematic reviews using ROBIS tool

= low risk;  = high risk;  = unclear risk

## Discussion

To determine the best available evidence and provide recommendations regarding the usage of drainage after primary total hip or knee arthroplasty, we conducted this summary of systematic reviews. After assessing the quality of methodology and risk of bias of included systematic reviews, we found two reviews conducted by Zan et al. for hip and Si et al. et al. for knee were selected as the best evidence, with highest AMSTAR score and low risk of bias.

Zan et al. [16] involved 12 RCTs assessing a total of 1498 patients and 1524 hips. The results indicated that whether to use closed-suction drainage had dual characters following the available evidence. They reported that the benefit of closed suction drainage included: decreased wound related complications, decreased patients of reinforcement and wound hematoma, less change in mid-thigh circumstance. However, the closed suction drainage prolongs the length of hospital stay and operation time, increases the amount of patients requiring for transfusion and the mean transfusion unit. Furthermore, no significant difference was found on blood loss, infection, volume of hematoma, deep venous thrombosis (DVT) and pulmonary embolism (PE) and the Harris score. In conclusion, they summarized that whether the closed-suction drainage is safe and effective was still filled with controversy, and appealed for more carefully and scientifically designed RCTs to further demonstrate the claim.

Si et al. [15] included 12 RCTs covering a total of 889 TKAs. They reported no significant differences in infection rate, blood loss, haematoma formation, DVT, postoperative VAS score or range of motion between the closed drainage and non-drainage TKAs. Ultimately, they concluded that there appears to be no clear benefit or drawback to the use of closed drainage after primary TKA. To provide better results, they suggest improving the use of closed drainage, such as temporary clamping, or combining it with late tourniquet release or tranexamic acid.

Parker et al. conducted the first systematic review about the usage of a closed suction drainage after an orthopedic surgery in 2001 [6] in Cochrane Library and updated in 2007 [8], they also published the results about the usage of a drainage after hip and knee arthroplasty in 2004 [7]. But they pooled all orthopedic surgery or hip and knee arthroplasty together, with results different from each single surgery, thus making the results not appropriate for the decision making for TKA and THA. Besides, they were all done more than 10 years ago, during which most perioperative management were poor compared to nowadays.

The systematic review published by Li et al. [13] showed low risk of bias (ROBIS) and highest quality (AMSTAR) in the present study, but was not chosen as the best evidence. The following reasons might count for it: (1) It only included the simultaneous bilateral TKA using the other side as control, but the subjects receiving simultaneous bilateral TKA are not the same in lots of aspects to the unilateral TKA patients, we can’t directly extend the conclusions to all primary TKA; (2) As they used the other knee of the same patients as control, it was inaccurate to compare total blood loss and transfusion rate. Based on the above interpretation, we only marked it as ‘Partial relevant’ in ROBIS phase 1.

Zhou et al. [10] demonstrated similar results with Zan, but concluded that the routine usage of drainage after THA may be of more impairment than benefit. Chen et al. [11] showed there is inadequate evidence to support usage of closed suction drainage after primary hip arthroplasty. However, this meta-analysis and the relevant studies had limitation such as poor trial methodology and inadequate report of outcomes. To intensify the evidence of results, further RCTs with larger number of testing cases and advanced methodology of patients, longer follow-up period and unified hip joint functional assessment are needed.

Zhang et al. conducted two systematic review in 2011 [9] and 2018 [17], with similar conclusion that the usage of closed suction drainage after TKA is probably not superior to no drainage for most outcome measures and therefore surgeons may wish to reconsider the routine usage of this empirical practice until there is further evidence. Quinn et al. [14] included only 6 studies and 4 outcomes, both the least of all systematic reviews, but the AMSTAR is not low.

Systematic reviews are commonly considered as the best way to supply highest level for decision making in clinical practice [25]. However, numbers of systematic reviews concerning the same topic have been published with conflicting conclusions. Thus, it is confused for decision makers to determine which to adopt regarding these treatment methods. The similar controversy also occurred concerning the usage of drainage after arthroplasty. Although numeral systematic reviews have been published involving this subject, there was still discordant conclusions. Such disparity makes it difficult for decision makers who rely on this synthesized evidence to help them decide whether to use a suction drainage after joint arthroplasty when the systematic reviews with pooled results are not unanimous.

To assess the methodological quality and critical appraisal of systematic reviews, the AMSTAR tool was applied in the present study. Furthermore, to collect the systematic reviews and evaluate the risk of bias, a newly developed ROBIS tool (www.robis-tool.info) was used. The best evidence was selected based on the Jadad decision algorithm, which provided a decision instrument concluding process for recognizing and settling reasons of discordance among systematic reviews. With the ultimate purpose to help policy-makers or clinicians to provide best evidence from discordant studies, and to apply best evidence into practice, it is well recognized for differencing among systematic reviews and with widely application [26–28]. Ultimately, two systematic reviews [15, 16] were selected in the present study with highest quality (AMSTAR), lower risk of bias (ROBIS), and providing the best evidence (Jadad decision algorithm).

Although the present study has several strengths, the following primary limitations could be considered: (1) Studies only in English language were included in the present study. It is possible that reviews written in non-English language have been omitted. (2) Several factors of primary trials, including study design, publication bias and clinical heterogeneity, might have impact on interpretation. Besides the study of Li evaluated only simultaneously bilateral TKA, all the other reviews included unilateral and bilateral surgery, THA and hemiarthroplasty, and different primary diagnosis for the surgery, but no one did a subgroup analysis about these variabilities. Some studies mentioned about factors that may affect the blood loss such as tourniquet usage, prosthesis type, TKA or THA usage, thromboprophylaxis, type of the device, duration used for drainage, clamping or not, but none were included into the pooled analysis. None of the studies included patients’ preference or satisfaction to make the decision. (3) Negative affluence on the level of evidence and cohesion of the pooled results will be posed because the systematic reviews assessed in the present study only included small volume RCTs without blinding.

According to the two mentioned conclusions from the selected systematic reviews, we therefore, do not have sufficient confidence to confirm the necessity and benefit of using a closed suction drainage after neither THA nor TKA. Exact recommendations cannot be developed based on the inconsistent evidence currently. Further studies are still required to verify the necessity and benefit of using a closed suction drainage after TKA and THA.

## Supplementary Information

**Additional file 1: Table 1.** Primary Studies Included in Previous Systematic Reviews.

**Additional file 2: Table 2.** Heterogeneity of each outcome in included Systematic Reviews.

## Data Availability

The data analyzed during the current study is available from the corresponding author on reasonable request.
