# Supplementary material for: The usage of drainage after primary total hip or knee arthroplasty: best evidence selection and risk of bias considerations
Source: BMC Musculoskelet Disord. 2021 Dec 8;22:1028. doi: 10.1186/s12891-021-04897-z (PMC8656000; doi:10.1186/s12891-021-04897-z)
Supplement: Supplementary file 1 — Additional file 1: Table 1. Primary Studies Included in Previous Systematic Reviews. [file 12891_2021_4897_MOESM1_ESM.docx]

**Table 1.** Primary Studies Included in Previous Systematic Reviews.

|  | Hip | | | |  | Knee | | | | |  | Hip and Knee | | |
| --- | --- | --- | --- | --- | --- | --- | --- | --- | --- | --- | --- | --- | --- | --- |
|  | Chen 2014 | Kelly 2014 | Zan 2016 | Zhou 2013 |  | Li 2015 | Quinn 2015 | Si 2016 | Zhang 2011 | Zhang 2018 |  | Parker 2001 | Parker 2004 | Parker 2007 |
| Adalberth et al. 1998 |  |  |  |  |  |  | + | + | + | + |  | + | + | + |
| Beer et al. 1991 |  |  |  | + |  |  |  |  |  |  |  | + | + |  |
| Cao et al. 2009 |  |  |  |  |  |  |  |  | + |  |  |  |  |  |
| Cheung et al. 2010 | + | + | + | + |  |  |  |  |  |  |  |  |  |  |
| Crevoisier et al. 1998 | + | + |  | + |  |  |  | + | + | + |  | + | + | + |
| de Andrade et al. 2010 |  |  |  |  |  |  |  |  |  | + |  |  |  |  |
| Dora et al. 2007 | + | + | + | + |  |  |  |  |  |  |  |  |  |  |
| Esler et al. 2003 |  |  |  |  |  |  | + | + | + | + |  |  | + | + |
| Fan et al. 2013 |  |  |  |  |  | + |  | + |  | + |  |  |  |  |
| Gonzalez Della Valle et al. 2004 | + | + | + | + |  |  |  |  |  |  |  |  |  | + |
| Hill 2003 |  |  |  | + |  |  |  |  |  |  |  |  | + |  |
| Holt et al. 1997 |  |  |  |  |  |  | + | + | + | + |  | + | + | + |
| Horstmann 2012 |  | + |  |  |  |  |  |  |  |  |  |  |  |  |
| Jenny et al. 2001 |  |  |  |  |  |  | + | + | + | + |  |  | + | + |
| Jhurani et al. 2016 |  |  |  |  |  |  |  |  |  | + |  |  |  |  |
| Johansson et al. 2005 | + |  |  | + |  |  |  |  |  |  |  |  |  | + |
| Kim et al. 1998 hip | + | + | + | + |  |  |  |  |  |  |  | + | + | + |
| Kim et al. 1998 knee |  |  |  |  |  | + | + | + | + | + |  |  | + | + |
| Kleinert et al. 2012 | + | + | + | + |  |  |  |  |  |  |  |  |  |  |
| Korkala 1999 |  |  |  |  |  |  |  |  |  |  |  | + |  |  |
| Leb et al. 1995 |  |  |  |  |  |  |  |  |  |  |  | + | + | + |
| Li et al. 2011 |  |  |  |  |  |  |  | + |  | + |  |  |  |  |
| Lin et al. 2009 |  |  |  |  |  |  |  |  | + |  |  |  |  |  |
| Liu et al. 2014 |  |  |  |  |  |  |  | + |  |  |  |  |  |  |
| Matsuda et al. 2007 | + |  | + | + |  |  |  |  |  |  |  |  |  |  |
| Mengal et al. 2001 | + |  |  |  |  |  |  |  | + | + |  | + | + | + |
| Murphy and Scott 1993 | + | + |  | + |  |  |  |  |  |  |  |  | + | + |
| Niskanen et al. 2000 | + | + | + | + |  |  |  | + | + | + |  | + | + | + |
| Nixon et al. 2000 |  |  |  |  |  |  |  |  |  |  |  |  | + | + |
| O’Brien 1997 |  | + |  | + |  |  |  |  |  |  |  |  |  |  |
| Omonbude et al. 2010 |  |  |  |  |  |  | + | + | + | + |  |  |  |  |
| Ovadia et al. 1997 | + | + | + | + |  |  |  | + | + | + |  | + | + | + |
| Ravikumar 2001 |  |  |  | + |  |  |  |  |  |  |  | + | + | + |
| Ritter et al. 1994 |  | + |  | + |  |  |  |  | + | + |  | + | + | + |
| Sharma et al. 2016 |  |  |  |  |  |  |  |  |  | + |  |  |  |  |
| Strahovnik et al. 2010 | + | + | + | + |  |  |  |  |  |  |  |  |  |  |
| Tao et al. 2006 |  |  |  |  |  |  |  |  | + | + |  |  |  |  |
| von Roth et al. 2012 |  | + | + | + |  |  |  |  |  |  |  |  |  |  |
| Walmsley et al. 2005 | + | + | + | + |  |  |  |  |  |  |  |  |  | + |
| Wang et al. 2016 |  |  |  |  |  |  |  |  |  | + |  |  |  |  |
| Watanabe et al. 2016 |  |  |  |  |  |  |  |  |  | + |  |  |  |  |
| Widman et al. 2002 | + | + |  | + |  |  |  |  |  |  |  |  | + | + |
| Xiong 2008 |  |  |  |  |  | + |  |  | + |  |  |  |  |  |
| Zeng et al. 2014 |  |  | + |  |  |  |  |  |  |  |  |  |  |  |
