# Supplementary material for: The usage of drainage after primary total hip or knee arthroplasty: best evidence selection and risk of bias considerations
Source: BMC Musculoskelet Disord. 2021 Dec 8;22:1028. doi: 10.1186/s12891-021-04897-z (PMC8656000; doi:10.1186/s12891-021-04897-z)
Supplement: Supplementary file 2 — Additional file 2: Table 2. Heterogeneity of each outcome in included Systematic Reviews. [file 12891_2021_4897_MOESM2_ESM.docx]

**Table 2.** Heterogeneity of each outcome in included Systematic Reviews.

|  |  |  | Hip | | | | | | |  | Knee | | | | | | | |
| --- | --- | --- | --- | --- | --- | --- | --- | --- | --- | --- | --- | --- | --- | --- | --- | --- | --- | --- |
|  |  |  | Chen 2014 | Kelly 2014 | Parker2001 | Parker2004 | Parker2007 | Zan 2016 | Zhou2013 |  | Li 2015 | Parker2001 | Parker2004 | Parker2007 | Quinn 2015 | Si 2016 | Zhang 2011 | Zhang 2018 |
| **complications** | Total complications | |  |  |  |  |  | 41.0% |  |  | 12.0% |  |  |  |  |  |  |  |
|  | Total complications without Erythema and Ecchymosis | |  |  |  |  |  |  |  |  | 59.0% |  |  |  |  |  |  |  |
|  | wound | wound hematoma | 0.0% | 39.0% | 0.0% | NM | 0.0% | 0.0% | 39.0% |  |  | 45.5% | NM | 0.0% |  | 0.0% |  |  |
|  |  | reinforcement of the dressing |  |  | 0.0% | NM | 28.1% | 70.0% | 77.0% |  |  | 35.6% | NM | 77.0% |  |  | 96.0% | 77.0% |
|  |  | Wound dehiscence or separation of the suture line |  |  | NA |  | NA |  |  |  |  | NA |  | 57.3% |  |  |  | 64.3% |
|  |  | persistent oozing | 0.0% |  | 0.0% | NM | 60.5% |  | 0.0% |  |  | 40.7% | NM | 4.0% |  | 35.0% |  |  |
|  |  | skin edge necrosis |  |  |  |  | NA |  |  |  |  |  |  | NA |  |  |  |  |
|  |  | Erythema of the wound |  |  |  |  | NA |  |  |  |  |  |  | NA |  |  |  |  |
|  | reoperation for the treatment of wound healing complications | |  |  | 27.0% | NM | 0.0% |  | 0.0% |  |  | NA | NM | NA |  |  |  |  |
|  | infection | Not classified | 0.0% | 0.0% | 38.1% | NM | 0.0% | 19.0% | 0.0% |  |  | 0.0% | NM | NA |  | 0.0% | 0.0% |  |
|  |  | superficial |  |  |  |  |  |  |  |  |  |  |  |  |  | 0.0% |  | 0.0% |
|  |  | deep |  |  |  | NM | NA |  |  |  |  |  | NM | NA |  | 0.0% |  | 0.0% |
|  | Limb | bruising or ecchymosis |  |  |  |  | 71.7% |  |  |  |  |  |  | 87.9% |  | 82.0% | 82.0% | 80.1% |
|  |  | swelling of the limb |  |  |  |  |  | 94.0% | 20.0% |  | 63.0% |  |  |  | 12.0% |  |  |  |
|  | DVT /PE | DVT /PE |  |  | NA | NM |  | NM |  |  |  | 8.0% | NM |  |  |  |  |  |
|  |  | DVT | 0.0% |  |  |  | 0.0% |  | 0.0% |  |  |  |  | 25.3% |  | 5.0% | 4.0% | 0.0% |
|  |  | PE |  |  |  |  | 0.0% |  | 0.0% |  |  |  |  | 0.0% |  |  |  |  |
|  | mortality |  |  |  |  |  | NA |  |  |  |  |  |  | NA |  |  |  |  |
| **Others** | Pain | not classified |  |  |  |  |  |  | 0.0% |  |  |  |  |  |  |  |  |  |
|  |  | PostOp Day 1 |  |  |  |  |  |  |  |  |  |  |  |  |  | 0.0% |  |  |
|  |  | PostOp Day 7 |  |  |  |  |  |  |  |  |  |  |  |  |  | 0.0% |  |  |
|  |  | PostOp Day 14 |  |  |  |  |  |  |  |  |  |  |  |  |  | 0.0% |  |  |
|  | Length of Stay | |  |  |  |  | 40.4% | 82.0% |  |  |  |  |  | 0.0% |  |  |  | 76.0% |
|  | Operative time | |  |  |  |  |  | NM |  |  |  |  |  |  |  | 8.0% |  |  |
| **Function** | ROM | Not classified |  |  |  |  |  |  |  |  |  |  |  |  |  |  | 17.0% |  |
|  |  | PostOp Day 2 |  |  |  |  |  |  |  |  |  |  |  |  |  | 47.0% |  |  |
|  |  | PostOp Day 3 |  |  |  |  |  |  |  |  |  |  |  |  |  | 89.0% |  |  |
|  |  | PostOp Day 7 |  |  |  |  |  |  |  |  |  |  |  |  | 0.0% | 19.0% |  | 28.2% |
|  |  | PostOp Day14 |  |  |  |  |  |  |  |  |  |  |  |  | 0.0% | 0.0% |  | 52.1% |
|  |  | PostOp month 3 |  |  |  |  |  |  |  |  |  |  |  |  |  |  |  | 7.3% |
|  |  | PostOp 1 year |  |  |  |  |  |  |  |  |  |  |  |  |  | 0.0% |  |  |
|  | Strength |  |  |  |  |  |  |  |  |  |  |  |  |  |  |  |  | 0.0% |
| **blood related** | transfusion | rate | 28.0% | 37.0% | NA | NM | 0.0% | 20.0% | 37.0% |  |  | 39.8% | NM | 11.6% |  | 43.0% | 63.0% | 52.3% |
|  |  | unit |  | 52.0% |  |  | NA |  | 79.0% |  |  |  |  | NA |  | 0.0% |  |  |
|  | Blood loss volume | intr-op |  |  |  |  |  | NM |  |  |  |  |  |  |  |  |  |  |
|  |  | Calculated total blood loss |  | 96.0% |  |  | NA | NM | 0.0% |  |  |  |  | NA |  | 90.0% |  | 87.4% |
|  |  | hidden blood loss |  |  |  |  |  |  |  |  |  |  |  |  |  | 93.0% |  |  |
|  | blood tests | Fall in Hematocrit |  |  |  |  | NA |  | NM |  |  |  |  | NA |  |  |  | 0.0% |
|  |  | PostOp Hemoglobin |  |  |  |  |  |  | NM |  |  |  |  |  |  |  |  |  |
|  |  | Fall in Hemoglobin Day 2 |  |  |  |  |  |  |  |  |  |  |  |  | 0.0% |  |  |  |

NM, not mentioned; NA, not available.
